# Bumblebee Abundance in Species-Rich Grasslands in Southern Sweden Decreases with Increasing Amount of Arable Land at a Landscape Level

**DOI:** 10.3390/insects15120982

**Published:** 2024-12-11

**Authors:** Per Milberg, Karl-Olof Bergman, Gabriella Fjellander, Malin Tälle, Lars Westerberg

**Affiliations:** IFM Biology, Conservation Ecology Group, Linköping University, 583 81 Linköping, Sweden

**Keywords:** *Bombus*, landscape, agriculture, field, spatial

## Abstract

Conservation of bumblebee populations is essential because of their role as pollinators. Declines in bumblebee abundance have been documented in recent decades, mostly attributed to agricultural intensification, landscape simplification and loss of semi-natural grasslands. In this study, we investigated the effects of landscape composition on bumblebee abundance at different spatial scales in 476 semi-natural grassland sites in southern Sweden. The area of arable land had a negative effect on total bumblebee abundance at all scales. This was most pronounced for short-tongued bumblebees, species typical of forested landscapes, and species with medium to large colony sizes and early queen emergence. The area of semi-natural grassland had a clear negative effect up to 1 km, affecting short-tongued bumblebees in both forested and agricultural landscapes and species with medium colony sizes and early queen emergence. The negative effect of arable land on bumblebees calls for action to adapt farming practices to ensure their conservation, e.g., by reducing pesticide use, improving crop diversity and promoting the presence of floral resources and alternative bumblebee habitats, such as species-rich field margins.

## Simple Summary

Declines in bumblebee abundance have been documented in recent decades, mostly attributed to agricultural intensification, landscape simplification and loss of semi-natural grasslands. We investigated the effects of landscape composition on bumblebee abundance at different spatial scales in 476 semi-natural grassland sites in southern Sweden. The area of arable land had a negative effect on total bumblebee abundance at all scales. This was most pronounced for short-tongued species, species typical of forested landscapes, and species with medium to large colony sizes and early queen emergence. The negative effect of arable land on bumblebees suggests that there is scope for improving their conservation, for example, by reducing pesticide use, improving crop diversity and promoting the presence of floral resources and alternative bumblebee habitats, such as species-rich field margins.

## 1. Introduction

Pollinators are essential in agricultural landscapes [1,2], as pollination increases the reproductive success of plants [3,4], with an estimated 35% of global crop production dependent on insect pollination [1]. In addition to human needs, pollinators are essential for wild flora. Wild pollinators, such as bumblebees (*Bombus* spp.), are particularly important, as they are often more efficient pollinators than the domesticated honeybee (*Apis mellifera*) [5,6].

Bumblebee abundance and diversity are declining worldwide [7,8,9], with shifts in bumblebee community composition, declines in several long-tongued bumblebee species and increased dominance of a few short-tongued species [10]. These declines can be explained by factors such as the introduction of alien species, pathogens and parasites, and climate change [11]. However, the main driver of pollinator decline is likely to be the intensification of agricultural practices [12]. In addition to increased pesticide use [12,13,14], this intensification has led to changes in landscape composition, with an increase in arable land and a loss of habitats important for bumblebees, such as semi-natural grasslands and forests [15,16,17,18,19,20]. These types of changes negatively affect bumblebees and other pollinators by impeding pollinator movements and reducing the availability of floral resources and nesting sites [20,21,22,23,24,25,26]. Effects of landscape composition may be scale-dependent [27], as suggested by previous studies on the effects of landscape composition on pollinators [21,28]. For example, bumblebees tend to be affected by habitat availability at larger spatial scales than solitary bees [21], probably reflecting differences in foraging time. In order to effectively conserve bumblebees, it is important to better understand how landscape composition affects them and how this varies at different spatial scales.

Therefore, the aim of this exploratory study was to investigate if and how bumblebee abundance in species-rich semi-natural grasslands is affected by land use in the surrounding landscape at different scales. In addition, we investigated whether effects varied between groups of bumblebees expected to be more or less sensitive to agricultural intensification. First, we examined whether effects differed between long-tongued bumblebees, which are expected to be more sensitive because they specialise in flowers with long corollas, and short-tongued species, which can be considered floral generalists because they use a wider range of floral resources [29]. Second, we investigated whether species adapted to forests are more sensitive to agricultural intensification than species using more open areas. Thirdly, we analysed the effect of differences in nest size between species, and, fourthly, the phenology of queen emergence.

## 2. Materials and Methods

### 2.1. Data Set

The data were collected as part of the environmental monitoring programme for valuable grasslands, which started in 2006 and is funded by the Swedish Board of Agriculture. This monitoring programme is coordinated with the National Inventory of Landscapes in Sweden (NILS [30]). The sample consisted of 631 landscape squares (25 km^2^) systematically distributed throughout Sweden and covering all landscape types. The grassland monitoring programme inventories were carried out on one or more species-rich grassland sites within these landscape squares. The grassland sites, 696 in total, were randomly selected from a database of valuable grasslands (“Ängs-och betesmarksinventeringen”) established by the Swedish Board of Agriculture in 2002–2004 [31,32,33].

In this monitoring programme, grassland sites were visited once over a five-year period (i.e., about 20% of sites were visited each year) and bumblebees were sampled once by trained personnel along non-overlapping transects. The sampling dates varied, mainly due to weather conditions; the median start and end dates over the five years were 22 June and 15 August, such that sampling occurred within the flight period of bumblebees in southern Sweden [34]. Bumblebees were recorded within two metres of the transect and identified to species level or, if not possible, to morphological groups [35]. Data on *Bombus lucorum* and *B. terrestris* were pooled because of the high risk of misidentification in the field [36].

During the inventory, flower abundance and sward height were estimated visually for each transect. Flower abundance was measured as the total cover of flowers of nectar-producing plants, and sward height was estimated as the percentage cover of sward vegetation within each of three height intervals: <5 cm, 5–15 cm and >15 cm [37].

The total area of different land use classes around each grassland site was estimated in 34 circles with different radii (from 100 to 10,000 m) per site using ArcGis (see [28], which uses the same land use data). Data on the location of semi-natural grassland were taken from the Swedish Board of Agriculture’s grassland inventory [37], while the location of arable land, forest and water was based on a map from the Swedish Land Survey (Lantmäteriet). Arable land, forest and water did not overlap, while semi-natural grassland occasionally overlapped with forest. We therefore decided to reduce the forest area by any possible overlapping semi-natural grassland. The scales were chosen to include not only normal foraging distances but also potential metapopulation patterns [28,38].

**Figure 1:**
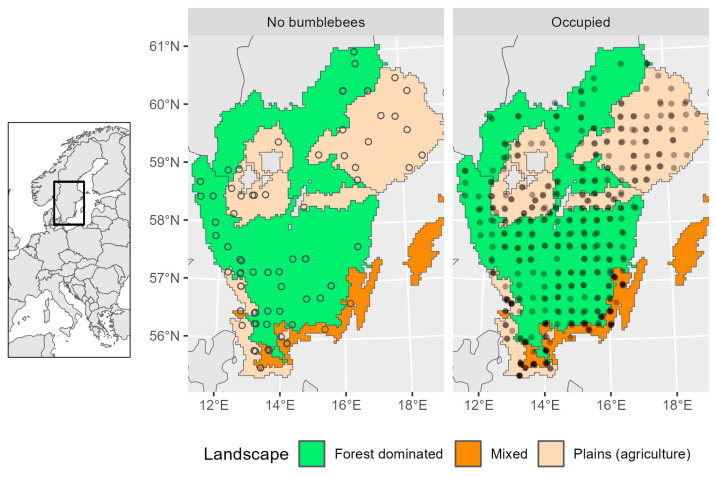
Map of the study area. The graph with open circles shows grasslands where no bumblebees were recorded, and the graph with filled circles shows all sites with occurrence of bumblebees.

The amount of semi-natural grassland, arable land, forest and water was measured in 34 concentric circles of 100 m to 10,000 m radius (i.e., scale) around each grassland (Figure 1). We used the database of the Swedish national survey of semi-natural meadows and pastures (TUVA) to extract the area of semi-natural grassland (habitat amount cover) for each scale. The grasslands included in the database are classified as high nature value [37] and consist of grazed or mowed semi-natural pastures and meadows and “grasslands that can be restored” (recently abandoned semi-natural grasslands). The database contains another class, “not applicable”, which was excluded from the present study. In order to measure the matrix composition around the semi-natural grassland sites, the Swedish Land Cover database (a Swedish version of the CORINE land cover database) was used to extract the amount of forest (coniferous, deciduous, mixed and young forest and clear-cuts combined), arable land and water areas surrounding the surveyed semi-natural areas at each scale. All land cover calculations and maps were made in ArcMap 10.1 (ESRI 2012). Other types of land cover were not used in the study.

**Figure 2:**
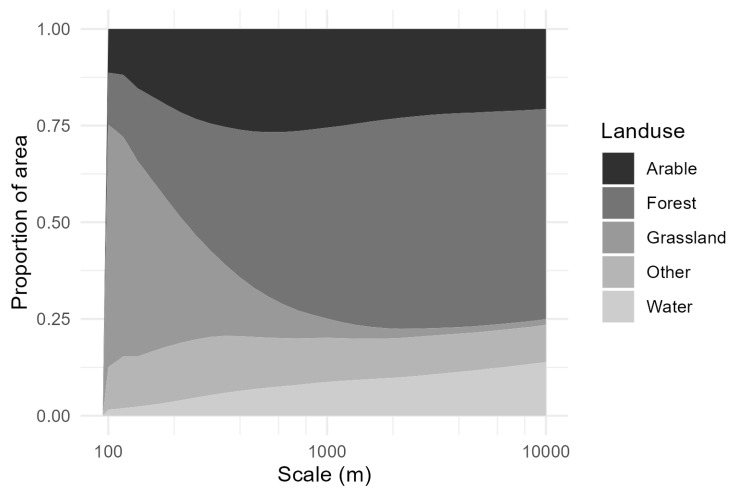
Percentages of land use types (semi-natural grassland, arable land, forest and water) at each spatial scale (radius of a circle around a site).

**Figure 3:**
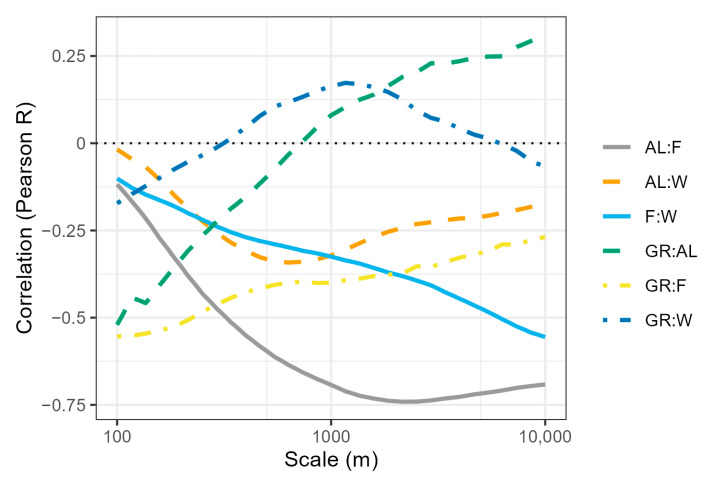
Correlation coefficients for each scale between pairs of land cover types (semi-natural grassland (GR), arable land (AL), forest (F) and water (W)).

The proportion of semi-natural grassland (mean area per grassland site) decreased from 63% of the total land use area at the smallest scale to 1% at the largest scale, arable land increased from 11% to 17% at the largest scale, forest increased from 14% to 51% at the largest scale and water also increased from 2% to 19% at the largest scale (Figure 2). Several of the land use classes were correlated, especially arable land and forest, which were strongly and negatively correlated at all scales (Figure 3).

### 2.2. Data Handling

The bumblebee data used were from a five-year inventory cycle (2007–2011). We restricted the study to southern Sweden (476 grassland sites in NILS strata 1–6) and excluded the islands of Gotland and Öland in order to obtain a relatively homogeneous area in terms of bumblebee species composition.

To investigate whether groups of bumblebees responded differently to land use, bumblebees were grouped on the basis of the following characteristics:

(i) Tongue length: long-tongued or short-tongued [39];
(ii) Habitat preference: agricultural or forested landscapes (www.artfakta.se);
(iii) Colony size: small, medium or large [40];
(iv) Queen emergence: early or late [40].

Species groups varied in terms of the number of species or specimens (Table 1), but the spatial cover was generally acceptable for analysis.

The bumblebee abundance for each grassland site (total abundance and abundance of each bumblebee group) was summed. Mean grass height per grassland site was calculated as a weighted average of the mean heights (2.5, 7.5 and 25 cm) and percent cover as weights. Area data for semi-natural grassland, arable land, woodland and water were square-root-transformed to reduce the influence of zero and high cover. Mean flower abundance was log10(x + 1)-transformed prior to analysis to obtain an even distribution of the data.

### 2.3. Statistical Analyses

#### 2.3.1. Generalised Linear Mixed Model

A generalised linear mixed model with a negative binomial distribution, a log link function and a log of transect length as offset was used to relate bumblebee abundance to the explanatory variables. Analyses were performed in R version 4.3.2 [41]. We used the R package glmmTMB [42] to model abundance in the species group as a function of areas of different land use, the weighted average of flowers and a random effect of 25 km^2^ squares nested in a year. The negative binomial was chosen because the responses were positive integers and because it can handle zero-inflated data. We also compared the AIC to models with, e.g., Gaussian and Poisson distribution functions, and chose ‘nbinom2’ as it had the lowest AIC.

Land use classes were correlated (Figure 3), which often led to collinearity in the models. The level of collinearity was tested using the vif function from the car package [43], with models without random factors fitted with the glm.nb model from the MASS package [44]. After removing forest and other land uses, the variance inflation was below 2 and acceptable; so, arable land, grassland, water and the weighted average of flowers were retained as fixed factors. The random factors were chosen because grasslands within a square were visited in the same year and bumblebee abundance varied between years. In this way, we controlled both for repeated observations of a square and year. Models with square and year as random factors had a lower AIC than simpler models.

To reduce the effects of spatial dependence by not using the same landscape data twice, we ensured that the buffer circles of the 476 grassland sites did not overlap. Non-overlapping sites were randomly selected at each scale. This was repeated a total of 100 times at each scale and the median z-value was calculated.

Model fit was assessed by residual analysis using the DHARMa package [45]. We fitted many models (one for each species group and scale: 310 models in total) and tested 40 models. All models tested had generally well-behaved residuals (i.e., they passed the KS test, the dispersion test and the outlier test), but eight models showed some deviations in the quantile test of the simulated residuals. After inspection, we found that the deviations were not systematic, and we argue that some deviations are to be expected due to the large number of tests. We accepted all models, rather than fitting several separate models, to facilitate comparison of the results.

We also tested for spatial autocorrelation in the residuals using the Moran’s I function implemented in the ape package [46] and a glm model with square, region and stratum as grouping variables. Autocorrelation was present in the majority of models for Moran’s I and glm with square as the explanatory factor, but glm with regions and stratum was rarely significant. This suggests that spatial autocorrelation was mostly local, at 25 km^2^. Another possibility is that model ‘shrinkage’ of estimates for squares led to unintended bias in the residuals: there were insufficient data for individual squares (usually one to four grasslands in a square), which caused estimates to shrink towards the population mean, leading to biased residuals at the square level. To further reduce the effects of spatial dependence, we ensured that the buffer circles of grassland sites did not overlap. Non-overlapping sites were randomly selected at each scale prior to model fitting. This was repeated a total of 100 times at each scale. Nevertheless, the majority of the models showed significant Moran’s I scores, and most of the 25 km^2^ square GLMs were significant. It appeared that these models did not adequately account for spatial autocorrelation, but a visual inspection of the geographical distribution showed that squares with high and low residuals occurred throughout the study area. Given the small-scale nature of the autocorrelation, the presence of many clusters and the non-overlapping buffer procedure, we argue that the results are at least not dominated by a few hotspots at larger scales.

#### 2.3.2. Potential Confounding Factors

Bumblebees can be influenced by site conditions such as grass height and flower abundance [47,48,49,50,51]. In addition, geographical location within the study area may reflect climate (e.g., decreasing precipitation towards the east and decreasing temperature towards the north). It is therefore reasonable to adjust for these factors when assessing the influence of land use in the surrounding landscape. A pre-analysis was therefore carried out using mean sward height, mean flower abundance, and east and north coordinates as ex post variables to determine whether any of these site-specific variables should be included in the analyses. Based on these pre-analyses, mean flower abundance was included as an explanatory variable in all analyses.

## 3. Results

A total of 3679 bumblebee individuals representing 26 species were recorded (Table 1). The two most common taxa were *Bombus lucorum/terrestris* (1521) and *B. pascuorum* (672), and thirteen of the species were rare (less than 1% of individuals recorded; Table 1).

**Figure 4:**
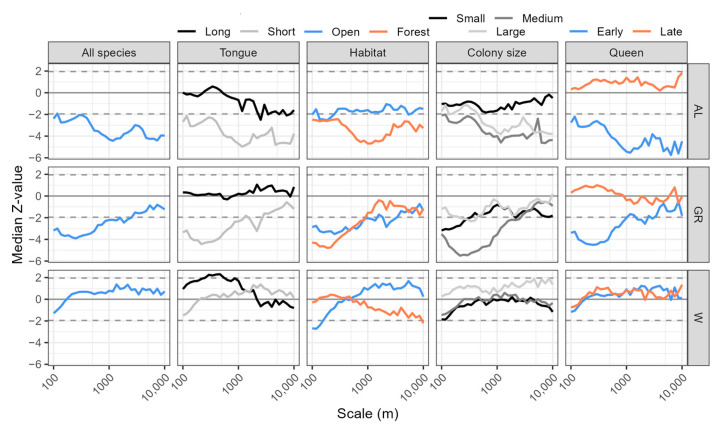
Medians of z-values from a model with total bumblebee abundance and different groups of bumblebees as a function of land use type at different spatial scales (GR: area of semi-natural grassland; AL: arable land; W: water). Groups of bumblebees were based on tongue (length: long-tongued or short-tongued), habitat (preference: open [agricultural] or forested), colony size (small, medium or large) and queen with respect to emergence date (early or late). Categories and sources are listed in Table 1. The dashed lines represent 0.05 levels of significance for positive and negative z-values. The z-values indicate the strength and direction of the relationships, with larger absolute values indicating stronger relationships and positive or negative signs indicating whether the relationship is positive or negative.

There was an overall clear negative effect of arable land area on total bumblebee abundance at all scales, and this effect increased at larger scales (Figure 4). The area of semi-natural grassland had a significant negative effect on bumblebee abundance up to about 1 km, but that effect disappeared at larger spatial scales. The effect of water on all species was non-significant over all scales.

There was also an overall negative effect of arable land area on short-tongued species at all scales, and this effect increased at larger scales. Long-tongued species were also negatively affected by arable land, but only at larger spatial scales. There was a negative effect of grasslands on short-tongued species at smaller spatial scales, and no effects of grasslands on long-tongued species. Water had overall small effects on both tongue-length groups but had a positive effect on long-tongued species at scales of a few hundred meters.

Forest species showed a more negative response to surrounding arable fields than species connected to open habitats. However, both groups showed similar responses to grasslands—a negative response at smaller spatial scales but a non-significant response at larger scales. Water had no effect on either group.

There was a negative effect of arable land area on bumblebees with medium and large colonies, especially at large scales. Bumblebees with small colonies showed no significant responses. However, bumblebees with small colonies were negatively affected by grasslands at small spatial scales. Bumblebees with medium colony sizes also showed a negative response to grasslands up to 1000 m, while species with large colonies showed no significant response. There was no response to amount of water in the surroundings for any colony size group.

There was a clear negative effect of arable land area on bumblebees with early-emerging queens, especially at large scales, while species with late-emerging queens showed no significant response. There was also a negative effect of grasslands on bumblebees with early-emerging queens at smaller spatial scales and no effect on species with late-emerging queens. There was no response to amount of water in the surroundings for either species groups with early- or late-emerging queens.

## 4. Discussion

### 4.1. Effect of Land Use Type

This study found that species-rich semi-natural grasslands surrounded by a larger area of arable land had lower total bumblebee abundance than those not surrounded by arable land. These results confirm conclusions from previous studies that landscape composition is an important factor influencing pollinator diversity and abundance [23,24,26] and that landscapes with more arable land negatively affect wild bees, butterflies and hoverflies [28,52,53,54].

The obvious interpretation of why grassland sites surrounded by arable land have low bumblebee abundance is that arable land is a low-quality habitat or non-habitat for bumblebees. Thus, landscapes with large areas of arable land reduce the amount of potential resources for bumblebee populations in grassland sites [55]. There are four non-exclusive hypotheses as to why arable land is located close to non-habitat. First, arable crops may not provide the wildflower resources (nectar and pollen) needed by bumblebees in sufficient abundance (e.g., [56]). Second, mass-flowering crops may attract and provide food resources for bumblebees, but only for a limited period of time and not throughout the entire flight season [57,58]. In addition, mass flowering crops may be rare compared to other crop types. In the current study, oilseed rape (*Brassica napus* and *Brassica rapa* ssp. *oleifera*; [59,60]) accounted for only 3–4% of the total area of arable land in Sweden in 2007–2011. Red clover seed leys are a very attractive crop for bumblebees [61,62], but on average only 2000 ha are currently cultivated per year [10]; for this two-year crop, only the harvest year involves mass flowering (only 0.04% of Swedish arable land). Thirdly, other potentially useful crops such as clover leys are cut so frequently that they provide limited resources. Fourth, arable land may be non-habitat due to the use of pesticides [14]. Pesticides such as neonicotinoids can lead to reduced bumblebee colony growth and reproduction [13], and these were still used in Sweden during the study period (2007–2011), before they were restricted in the European Union in 2013 [13,63]. When interpreting the results, the possibility of a concentration/dilution effect on bumble bees in land use classes should also be considered [64].

As semi-natural grassland is such an important habitat for bumblebees and other pollinators [15,17], it is surprising that the area of grassland in the landscape generally had no positive effect on bumblebee abundance. Before considering why, it is worth noting that the area of grassland sampled was partially included in the statistical model (which adjusted for transect length). At least at smaller spatial scales, this may have eliminated an expected positive effect of grassland. However, as the total area of semi-natural grassland in the landscape was generally so small (at least compared to the area of arable land), any changes in the area of grassland had only a marginal effect. Alternatively, the negative effect of arable land masks any positive effect of grassland [65]. In addition, as the surveys were conducted on grassland sites, it may be that bumblebees have sufficient grassland available within the site itself to be less affected by the availability of grassland in the landscape. Finally, small, isolated grasslands may have more bumblebees per area than expected due to a concentration effect [64], an effect that could mask the expected positive effect of grassland area.

#### Group-Wise Analyses

The stronger negative effect of arable land on short-tongued compared to long-tongued bumblebee species was surprising—at least if one considers that long-tongued species are specialists and therefore more vulnerable to increasing arable land [29]. One reason for this finding is that sampling was carried out in semi-natural grasslands—often a preferred habitat for bees—which may buffer changes and patterns compared to most random sampling points in the landscape. Another reason why species that are more likely to be specialists were found to be less affected by the area of arable land may be that these species had already declined in response to landscape changes prior to the study period. Thus, they are currently in a period of stability at a low level (the so-called “ski-jump effect” [66]), while the increase in arable land is now so large that generalist species have started to decline. This has been suggested as a reason why rare invertebrate species are not declining more than common species in UK nature reserves [67].

Although we analysed groups of species, it is clear that species contributed differently to the observed patterns (the dominant *B. lucorum/terrestris* making up 41% of the bumblebees).

### 4.2. Effect of Scale

It is worth noting that the negative effects of arable land, which increased with scale, suggest that it reduces population densities (e.g., at the scale of foraging trips from a nest) as well as metapopulation dynamics (at much larger scales, e.g., [68]).

The current study showed that the effects of land use differed between spatial scales, highlighting the need to consider multiple scales when spatially planning conservation actions such as restoration (e.g., [28,38,69]). The greater negative effect of arable land at larger scales (Figure 4) may be due to the lack of floral resources in structurally simple landscapes with large open fields. It is clear that field boundaries or non-crop habitats between fields [21,57] have not compensated for the general lack of resources or increased mortality from pesticides in arable fields. Another reason may be that the data used in the current study were from a grassland inventory, such that the bumblebees always had some semi-natural grassland—an important habitat for bumblebees—nearby. If total bumblebee abundance had been measured in several different habitat types rather than one specific habitat, arable land might have had a greater effect, even at smaller scales [57]. It is worth pointing out that the negative effects of arable land, which increased with scale, suggest that it reduces population densities (e.g., at the scale of foraging trips from a nest) as well metapopulation dynamics (at much larger scales, e.g., [68]).

## 5. Conclusions

The present study found that bumblebee abundance decreased as the area of arable land in the landscape increased, especially at larger scales, e.g., in regions dominated by agriculture. This suggests that arable land may be a suboptimal or even non-habitat for bumblebees, possibly due to scarce floral resources and the use of pesticides in crop fields. As large-scale reductions in agricultural land use are unlikely, the best way to conserve bumblebee populations may be to adapt existing agricultural practices. At the landscape scale, this may include organic farming [70,71], occasional abandonment of chemical weed control, reduced use of pesticides and growing a diversity of crop species [23]. More complex landscapes with more and different floral resources and potential nesting sites can also benefit bumblebees, e.g., landscapes with smaller arable fields and the presence of flower strips, field margins and hedgerows with species-rich vegetation [72,73,74]. Increasing the habitat quality of individual grassland sites, e.g., by ensuring a high diversity and abundance of floral resources, may also be a way of reducing the negative effects of surrounding arable land use on bumblebees [48,50].

The sampling in this study was limited to grassland sites, but to better understand the influence of landscape composition on bumblebee communities, it would be desirable to extend the monitoring programme to other land use types. This could give a more detailed insight into how different habitats are used by bumblebee species with different characteristics.

## Figures and Tables

**Table 1:** Species of bumblebees (*Bombus* spp.) and numbers of individuals observed in the 476 grassland sites, and the species traits used to group them in the analyses.

*Bombus* Species	Number of Specimens	Tongue Length (Proboscis < 8 mm: Short) ^1^	Habitat Preference ^2^	Colony Size ^3^	Queen Emergence ^3^
Non-parasitic spp.					
*B. distinguendus*	2	Long	Open	Nd	Nd
*B. hortorum*	116	Long	Forest	Medium	Late
*B. humilis*	60	Long	Open	Nd	Nd
*B. hypnorum*	165	Short	Open	Medium	Early
*B. jonellus*	65	Nd	Forest	Small	Late
*B. lapidarius*	252	Short	Open	Large	Early
*B. lucorum & B. terrestris*	1521	Short	*	Large	Early
*B. lucorum/terrestris/sporadicus/soroeensis*	28	Short	*	*	*
*B. muscorum*	5	Short	Open	Small	Late
*B. pascuorum*	672	Short	Open	Medium	Early
*B. pratorum*	298	Short	Forest	Small	Early
*B. ruderarius*	65	Nd	Open	Small	Late
*B. soroeensis*	69	Short	Open	Medium	Late
*B. sporadicus*	2	Short	Forest	Nd	Nd
*B. subterraneus*	13	Long	Open	Medium	Late
*B. sylvarum*	224	Short	Open	Small	Late
Parasitic spp.					
*B. barbutellus*	7	Nd	Forest	Nd	Nd
*B. bohemicus*	45	Nd	Forest	Nd	Nd
*B. campestris*	18	Nd	Forest	Nd	Nd
*B. norvegicus*	2	Nd	Forest	Nd	Nd
*B. quadricolor*	4	Nd	Forest	Nd	Nd
*B. rupestris*	24	Nd	Open	Nd	Nd
*B. sylvestris*	21	Nd	Forest	Nd	Nd
*B. vestalis*	1	Nd	Open	Nd	Nd
Sum of Specimen	3679	Long: 191 Short: 3236	Open: 1552 Forest: 578	Small: 657 Medium: 1035 Large: 1773	Early: 2908 Late: 557

1: [39]. 2: www.artfakta.se (accessed on 15 March 2024). 3: [40]. * indicates cases where species differed within a group (e.g., one species prefers forest while the other prefers open habitat). Nd means no data available in the source used.

## Data Availability

The data used were from public databases. The modified data used in the analyses are available on request.
